# SRC/ABL inhibition disrupts CRLF2-driven signaling to induce cell death in B-cell acute lymphoblastic leukemia

**DOI:** 10.18632/oncotarget.25089

**Published:** 2018-05-01

**Authors:** Jolanda Sarno, Angela M. Savino, Chiara Buracchi, Chiara Palmi, Stefania Pinto, Cristina Bugarin, Astraea Jager, Silvia Bresolin, Ruth C. Barber, Daniela Silvestri, Shai Israeli, Martin J.S. Dyer, Giovanni Cazzaniga, Garry P. Nolan, Andrea Biondi, Kara L. Davis, Giuseppe Gaipa

**Affiliations:** ^1^ Department of Pediatrics, Bass Center for Childhood Cancer and Blood Disorders, Stanford University, Stanford, CA, USA; ^2^ M. Tettamanti Research Center, Pediatric Clinic, University of Milano Bicocca, Monza, Italy; ^3^ Cancer Research Center, Sheba Medical Center, Ramat Gan, Israel; ^4^ Laboratory of Onco-Hematology, Department of Women’s and Children’s Health, University of Padova, Padova, Italy; ^5^ Leicester Drug Discovery & Diagnostic Centre, University of Leicester, Leicester, United Kingdom; ^6^ Biostatistics and Clinic Epidemiology Center, University of Milano Bicocca, Monza, Italy; ^7^ Ernest and Helen Scott Haematological Research Institute, University of Leicester, Leicester, United Kingdom; ^8^ Baxter Laboratory in Stem Cell Biology, Department of Microbiology and Immunology, Stanford University, Stanford, CA, USA; ^9^ Department of Pediatrics, ASST-Monza, Ospedale San Gerardo/Fondazione MBBM, Monza, Italy

**Keywords:** acute lymphoblastic leukemia, cell signaling, signal transduction inhibitors, mass cytometry, minimal residual disease

## Abstract

Children with B-cell precursor acute lymphoblastic leukemia (BCP-ALL) overexpressing the *CRLF2* gene (*hiCRLF2*) have poor prognosis. CRLF2 protein overexpression leads to activated JAK/STAT signaling and trials are underway using JAK inhibitors to overcome treatment failure. Pre-clinical studies indicated limited efficacy of single JAK inhibitors, thus additional pathways must be targeted in *hiCRLF2* cells. To identify additional activated networks, we used single-cell mass cytometry to examine 15 BCP-ALL primary patient samples. We uncovered a coordinated signaling network downstream of CRLF2 characterized by co-activation of JAK/STAT, PI3K, and CREB pathways. This CRLF2-driven network could be more effectively disrupted by SRC/ABL inhibition than single-agent JAK or PI3K inhibition, and this could be demonstrated even in primary minimal residual disease (MRD) cells. Our study suggests SCR/ABL inhibition as effective in disrupting the cooperative functional networks present in *hiCRLF*2 BCP-ALL patients, supporting further investigation of this strategy in pre-clinical studies.

## INTRODUCTION

B-cell precursor acute lymphoblastic leukemia (BCP-ALL) is a heterogeneous disease resulting from the accumulation of genetic alterations in B-lymphoid precursor cells and represents the most common malignant disease in childhood [1, 2]. Although the pediatric 5-year survival rate now exceeds 85%, the survival following relapse still remains poor [3, 4]. Gene alterations involving the *CRLF2* (Cytokine Receptor Like Factor 2) gene are frequently present in high-risk BCP-ALL patients [5] as well as T-ALL [6] and result in overexpression of CRLF2 subunit of the heterodimeric receptor of TSLP (thymic stromal lymphopoietin), known as TSLPR [7].

Overexpression of *CRLF2* is present in up to 15% of high risk BCP-ALL patients [5] and 50% of both Down Syndrome–associated BCP-ALL and Ph-like BCP-ALL patients [8-10]. Subsets of CRLF2-overexpressing cells have been shown to also harbor activating mutations in *JAK, IL7R and NRAS* [11], as well as deletions of the *IKZF1* gene [12, 13], which similarly confer poor clinical prognosis [14]. Since these patients respond poorly to standard chemotherapy regimens, there is need to improve our understanding of the biology of this BCP-ALL subtype to devise new therapeutic approaches.

The important role played by *JAK2* and *IL7R* alterations in TSLPR downstream signaling of murine pro-B Ba/F3 has been widely investigated by several groups [7, 15, 16]. As previously demonstrated, alterations in *CRLF2* and/or *JAK2* are responsible for increased TSLP-dependent activation of JAK2, STAT5, and rpS6 phospho-species, suggesting that targeting these molecules may be a valid therapeutic option for these patients [17, 18]. The JAK1/2 inhibitor (i), ruxolitinib, is currently employed in a phase II clinical trial study of Ph-like ALL patients bearing *CRLF2* alterations (ClinicalTrials.gov Identifier: NCT02723994). However, Weigert *et al.* and Scheartzman *et al.* demonstrated limited *in vitro* efficacy of ruxolitinib in human BCP-ALL *CRLF2* rearranged (r)/*JAK2* mutated cell lines [19-21], suggesting that other pathways may be involved in TSLPR signaling and that treatment with ruxolitinib alone may not be sufficient for *hiCRLF2* patients, as also recently described by Tasian et *al*. [22].

To better understand the complexity and impact of CRLF2 overexpression, we utilized single-cell mass cytometry (CyTOF) [23] to examine the signaling networks downstream of the TSLPR in primary diagnostic *hiCRLF2* BCP-ALL bone marrow samples. CyTOF enabled examination of multiple signaling pathways simultaneously and we identified a network involving JAK/STAT, PI3K and CREB pathways activated in *hiCRLF2* patients. Perturbation of cells with inhibitors of the downstream TSLPR pathways, including a monoclonal antibody against the CRLF2 subunit, revealed the dual SRC/ABL inhibitor, dasatinib, to be effective in disrupting this network *ex vivo* and in inducing cell death to a similar degree as with the combination of JAK and PI3K inhibition. To determine if this network was relevant in drug resistance in *hiCRLF2* patients, we examined minimal residual disease (MRD) samples and observed the same network present at the time of diagnosis in these patients. Further, in two of three patients classified as poor responders, cells harboring this network phenotype were enriched at Day 8 and Day 15 time-points, suggesting that this network may be important in the early persistence of leukemic cells. Thanks to this single-cell analysis, we uncovered distinct and clinically-relevant signaling nodes that can be successfully targeted by using a dual SRC/ABLi both in diagnostic and MRD cells, suggesting new therapeutic perspectives for patients with BCP-ALL bearing *CRLF2* alterations.

## RESULTS

### TSLP stimulation induces simultaneous activation of multiple signaling pathways in *hiCRLF2* BCP-ALL primary samples

Single cells from twelve BCP-ALL primary diagnostic bone marrow samples, 6 *hiCRLF2* and 6 *loCRLF2*, were analyzed by CyTOF using a 39-antibody panel (Supplementary Table 1) to determine activated downstream pathways of CRLF2 in primary cells. Blast cells were gated as shown in Supplementary Figure 1A-1B and *CRLF2* over-expressing cells were faithfully identified by the mass cytometry platform as shown in panel A.

*HiCRLF2* patients demonstrated higher basal levels of pSTAT5 in the leukemic blasts compared to *loCRLF2* samples (mean 0.27 *vs.* 0.07, respectively) consistent with previous data [24], although not reaching statistical significance (p=0.0842). This higher basal pSTAT5 level is expected considering that our *hiCRLF2* cohort contained two patients bearing mutations in *JAK2* (Pt #2: R683G *JAK2* mutation and Pt #1 a novel *JAK2* insertion, L681-I682 insGL, in exon 16; see Table 1). No additional phosphoproteins were significantly different between *hiCRLF2* and *loCRLF2* samples in the basal state (data not shown).

**Table 1:** Main clinical and biological features of analyzed patients

Patients	Age at diagnosis (years)	Sex	WBC (x10^3/μL)	Down syndrome	Immuno-phenotype	MRD risk	Final risk	CRLF2 expression (FCM)	CRLF2 rearrangements (PCR/FISH)	JAK2 alterations (HRM)	IL-7 alterations	Ph-like (GEP)	Other genetic lesions (MLPA)
#1	15	M	1870	yes	B-II	HR-SER	HR	positive	P2RY8-CRLF2	L681-I682 ins GL	ex 6:T244I	no	del CDKN2A/2B, del IKZF1, del PAX5
#2	12	M	193750	no	B-II	IR	IR	positive	IGH@-CRLF2	R683G	wt	yes	n.a.
#3	4	F	108500^*^	yes	B-III	IR	HR	positive	P2RY8-CRLF2	wt	ex 5:S185C	no	n.a
#4	17	F	87210	no	B-III	SR	SR	positive	P2RY8-CRLF2	wt	wt	yes	del CDKN2A/2B, del IKZF1, del PAX5
#5	2	M	23940	no	B-II	SR	SR	positive	P2RY8-CRLF2	wt	wt	yes	wt
#6	12	F	53640	no	B-II	IR	HR	positive	Not known	wt	wt	yes	wt
#7	6	F	4200	no	B-II	IR	SR	negative	wt	wt	wt	n.a	del CDKN2A/2B, del IKZF1
#8	4	M	12650	no	B-II	HR-SER	HR	negative	wt	wt	wt	n.a	del ETV6, del BTG1
#9	4	F	25030	no	B-II	HR-SER	HR	negative	wt	wt	wt	n.a	del ETV6, del EBF1
#10	4	F	34800	no	B-II	IR	IR	negative	wt	wt	wt	n.a	del CDKN2A/2B, del IKZF1, del PAX5
#11	2	F	21950	no	B-II	IR	IR	negative	wt	wt	wt	n.a	wt
#12	16	F	6110	no	B-II	IR	IR	negative	wt	wt	wt	n.a	del PAX5, del ETV6, del RB1
#13	17	M	203000	no	B-II	HR-SER	HR	positive	IGH@-CRLF2	wt	n.a.	yes	wt
#14	4	F	1660	yes	B-II	IR	IR	positive	P2RY8-CRLF2	wt	n.a.	no	del PAX5
#15	4	M	5890	no	B-II	IR	HR	positive	P2RY8-CRLF2	wt	n.a.	yes	wt

^*^This count was performed in the bone marrow.

Abbreviations: M, male; F, female; wt, wild type; n.a., not available; del, deletion, HR, high risk; IR, intermediate risk; SR,standard risk; SER, slow early responder, FCM, Flow Cytometry; PCR, Polymerase Chain Reaction; FISH, Fluorescence In Situ Hybridization; HRM, High Resolution Melting, GEP, Gene Expression Profile; MLPA, Multiplex Ligation-dependent Probe Amplification.

**Figure 1:**
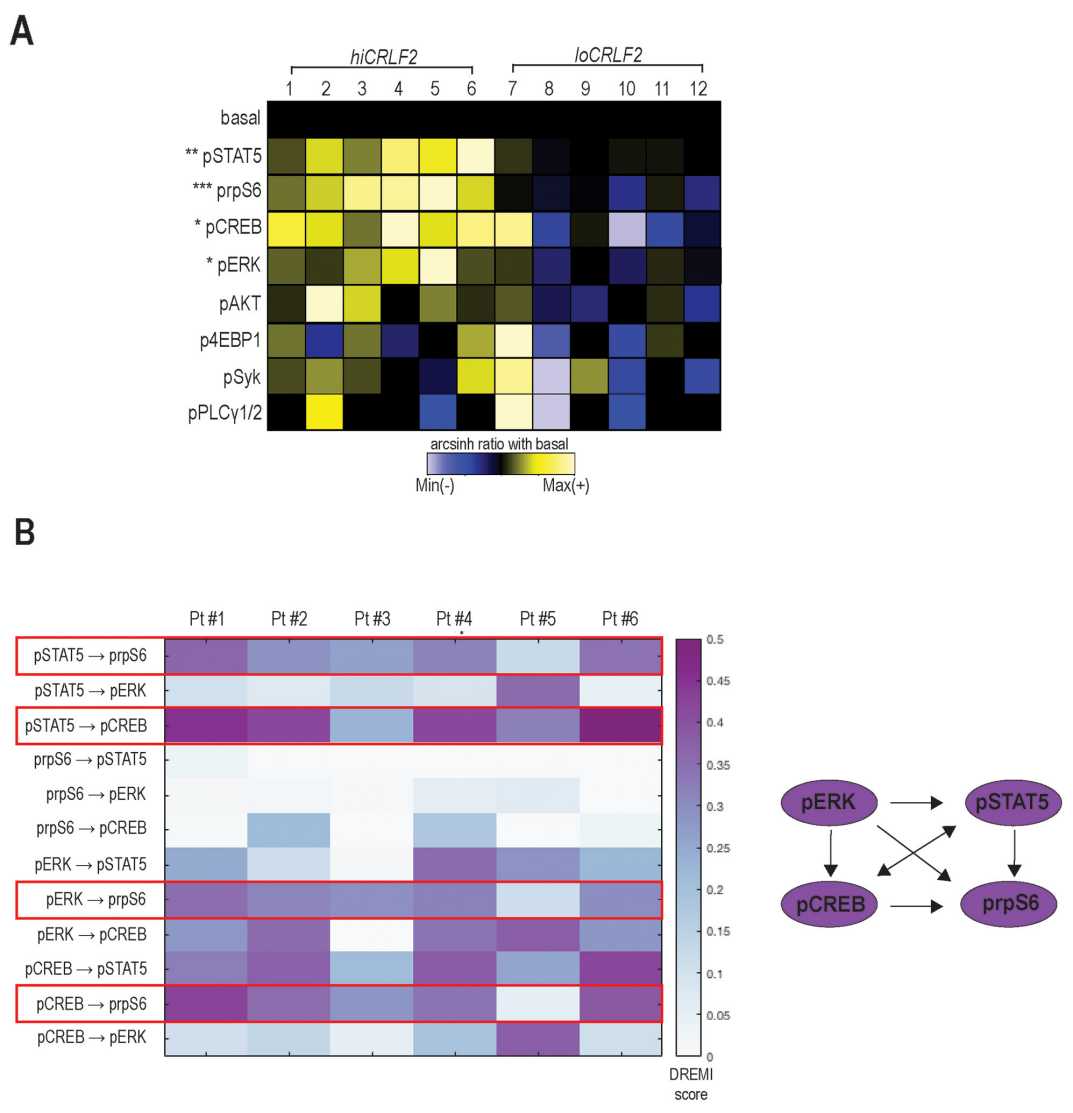
TSLP stimulation induces simultaneous activation of multiple signaling pathways in *hiCRLF2* BCP-ALL primary samples **(A)** Overview of TSLP-induced signaling in blast cells (gated as shown in Supplementary Figure 1) from BCP-ALL primary samples (column 1 - 6 *hiCRLF2* patients; column 7 - 12 *loCRLF2* patients). Each row represents the arcsinh ratio of a phosphoprotein in TSLP-treated cells over baseline levels from unstimulated cells (decreased phosphorylation (blue) versus increased phosphorylation (yellow) compared to their basal level). Asterisks indicate statistically significant differences between *hiCRLF2* and *loCRLF2* phosphoproteins, calculated by using an unpaired two-sided student’s t test (^*^ p<0.5, ^**^ p<0.01, ^***^ p<0.001). **(B)** Heatmap of the DREMI scores summarizing the signaling connections present within the TSLP-activated phosphoproteins in the *hiCRLF2* patients cohort. The red boxes highlight the strongest connections that are also reported in the diagram on the left.

Yet, *in vitro* stimulation with TSLP increased the phosphorylation levels of both STAT5 and rpS6 in *hiCRLF2* compared to *loCRLF2* cells (p=0.0054 and p=0.0006, respectively) (Figure 1A), as previously described [18]. Furthermore, we observed TSLP-induced phosphorylation of ERK and CREB in *hiCRLF2* cells but not in *loCRLF2* cells (pERK arcsinh ratio 0.09 *vs.* -0.01, p=0.0313; pCREB arcsinh ratio 0.15 *vs.* -0.04, p=0.0260, respectively) supporting the hypothesis that multiple pathways are involved in CRLF2-driven signaling.

To understand if the TSLP-activated molecules were activated as part of a network and to quantify the strengths of these relationships, we used conditional-Density Resampled Estimate of Mutual Information (DREMI) to reveal pairwise signaling interactions between molecules and conditional-Density Rescaled Visualization (DREVI) to visually render this function as a rescaled heatmap, as previously described [25].

DREMI analysis suggested pSTAT5, pCREB, pERK and prpS6 to be part of the same TSLP-induced signaling network in *hiCRLF2* patients (Figure 1B and Supplementary Figure 2A, red box) and, as expected, this was not detected in the *loCRLF2* patients (Supplementary Figure 2A, green box). Specifically, as cells activate pSTAT5 there is a concomitant activation of prpS6 and pCREB (Figure 1B, red boxes). In only one patient (Pt#5), prpS6 demonstrated high basal activation that was not dependent on pSTAT5 or pCREB, although the dependency between pSTAT5 and pCREB was maintained (Supplementary Figure 2B). We also found strong connections between pCREB and prpS6 as well as between pERK and prpS6 (Figure 1B, red boxes) supporting a downstream role of prpS6 in the TSLP signaling network. Thus, *hiCRLF2* cells demonstrate TSLP-dependent activation in JAK/STAT and prpS6 pathways but this single cell analysis revealed additional activated targets which together lie in a coordinated network.

### SRC/ABLi inhibition targets coordinated TSLP-induced signaling

**Figure 2:**
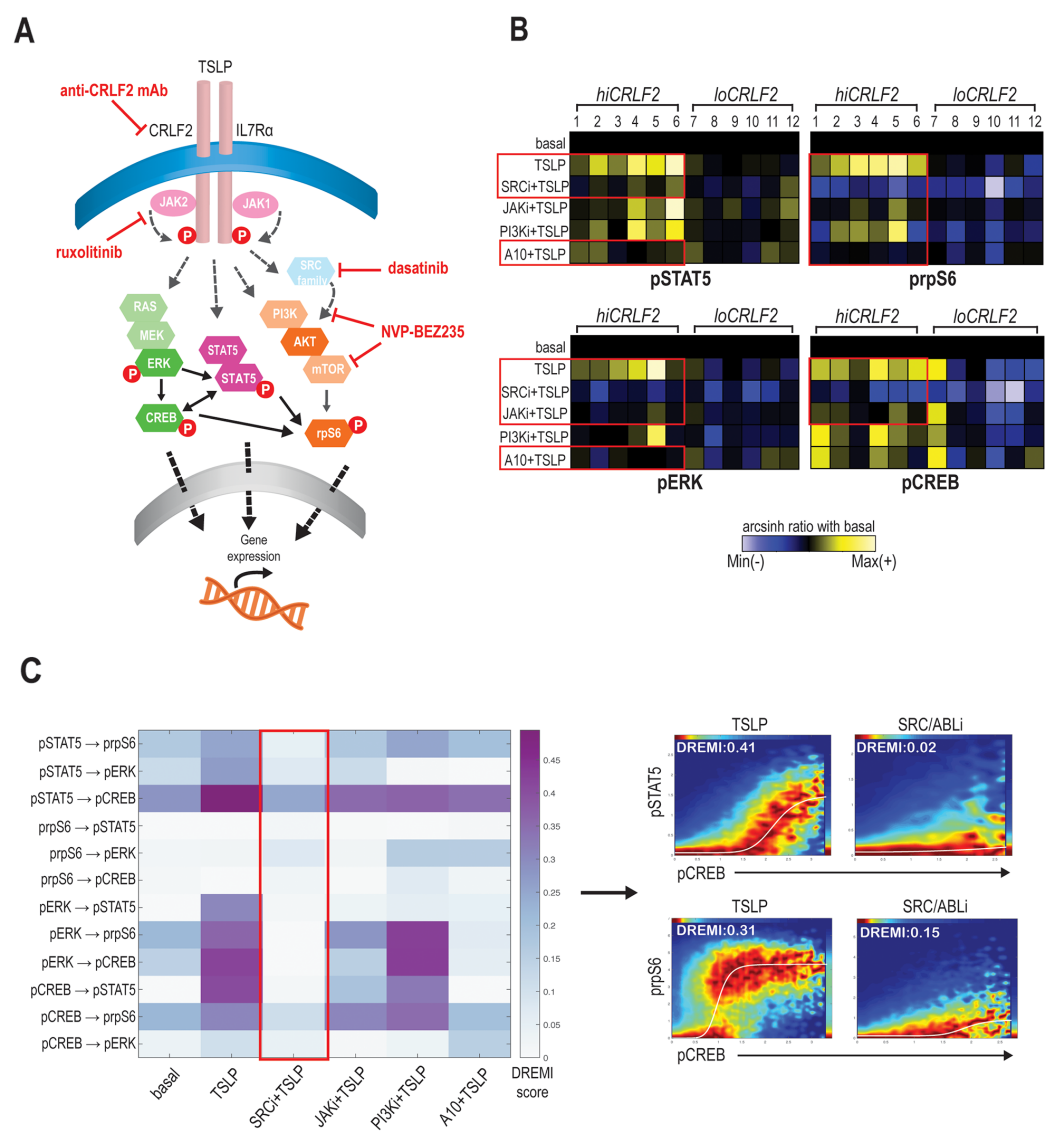
SRC/ABL inhibition targets coordinated TSLP-induced signaling **(A)** Schematic representation of TSLPR heterodimer signaling transduction in childhood *hiCRLF2* BCP-ALL and agents used in this study to target activated signaling nodes. The proteins not included in our CyTOF panel and the signaling connections not tested by DREMI are shadowed and grey. **(B)** Effects on TSLP-activated phosphoproteins (pSTAT5, prpS6, pERK and pCREB) in *hiCRLF2* and *loCRLF2* BCP-ALL primary cells following short-term single agent kinase inhibition. Each row represents a treatment condition and each phosphoprotein is showed as the arcsinh ratio of treated (TSLP or agent+TSLP) condition over baseline levels. **(C)** Overview of the DREMI scores across treatment conditions in the *hiCRLF2* cohort. The red box highlights the effect of SRC/ABLi in disrupting all the pairwise signaling relationships present within the 4 activated phosphoproteins comprising the TSLP-driven network. Arrow directs to DREVI visualization of the disruption of TSLP dependencies between pCREB → pSTAT5 and pCREB → prpS6. SRCi=dasatinib, JAKi=ruxolitinib, PI3Ki=NVP-BEZ235, A10=A10 anti-CRLF2 mAb.

To better understand the coordinated CRLF2 signaling connections and how to disrupt them, we subjected the same BCP-ALL primary samples to pharmacologic inhibition using kinase inhibitors (KIs) targeting JAK/STAT (ruxolitinib), SRC/ABL (dasatinib), or PI3K/mTOR (NVP-BEZ235) pathways (as represented in Figure 2A). In addition, we also employed an anti-CRLF2 mAb directed against the CRLF2 subunit of the TSLPR receptor.

We tested three mAbs (clones A10, A11 and G7) for their binding strength and signaling inhibition in *CRLF2*-rearranged human BCP-ALL cell line (MUTZ5) and in Ba/F3-hTSLPR expressing cells. Although all clones could specifically bind the CRLF2 subunit in both cell lines (Supplementary Figure 3A), only the A10 anti-CRLF2 mAb could strongly inhibit the TSLP-dependent activation of both pSTAT5 and prpS6 in MUTZ5 cells (p< 0.0001) and of pSTAT5 in Ba/F3-hTSLPR cells (p<0.0001; Supplementary Figure 3B), thus for further experiments we moved ahead with the A10 clone.

Exposing the primary BCP-ALL leukemic cells (either *hiCRLF2* or *loCRLF2*) to these treatments (Supplementary Table 2) and comparing phosphoprotein levels to the TSLP-stimulated condition, we found that the JAKi, ruxolitinib, decreased the TSLP-induced STAT5 activation in *hiCRLF2* samples but the response was heterogeneous (p=0.3338) as patients #1, #2 (both JAK2 altered) and patient #6 demonstrated a strong pSTAT5 inhibition with ruxolitinib. However, pSTAT5 was more efficiently and consistently inhibited by the SRC/ABLi, dasatinib, (p=0.0254) or by the A10 anti-CRLF2 mAb (p=0.0357) (Figure 2B, upper left, red boxes). These agents could also inhibit the TSLP-dependent activation of prpS6 in *hiCRLF2* samples as well, with dasatinib actually lowering prpS6 below basal levels (Figure 2B, upper right) (p<0.0001 for dasatinib and p=0.0009 for A10 anti-CRLF2 mAb). Again, prpS6 response to JAKi was sample-dependent as patients #1, #2 and patient #6 showed the best response (p=0.0038). Similarly, PI3K/mTOR inhibition inhibited the TSLP-mediated activation of prpS6 (p=0.0054).

TSLP-mediated activation of pERK and pCREB in *hiCRLF2* samples was consistently inhibited by the SRC/ABLi (Figure 2B, see red boxes lower left and right) (p=0.0039 and p<0.0001, respectively), as well as against basal pERK and pCREB in *loCRLF2* patients. ERK activation was decreased by JAKi and A10 anti-CRLF2 mAb (p=0.0404 by both agents), but activated CREB was only inhibited by the JAKi (p=0.0338) supporting previous evidence of active crosstalk within the pathways.

In our diagnostic cohort, of the 6 *hiCRLF2* patients, 4 were Ph-like (Pt#2, 4, 5 and 6) based on gene expression signature (see Table 1 and Supplementary Materials). In these Ph-like samples, there was more effective inhibition of pSTAT5 and pCREB by dasatinib compared to those who were non Ph-like (p=0.0285 and p=0.0388 respectively), however this result requires further investigation due to the small number of samples tested here.

By examining how these treatments affect the entire TSLP signaling network (pSTAT5, pCREB, pERK and prpS6) we found that, although all the agents were able to affect single phosphoproteins, the only treatment disrupting all their interactions was the SRC/ABLi, dasatinib, as shown in Figure 2C (red box).

### Signaling heterogeneity exists within *hiCRLF2* cells

**Figure 3:**
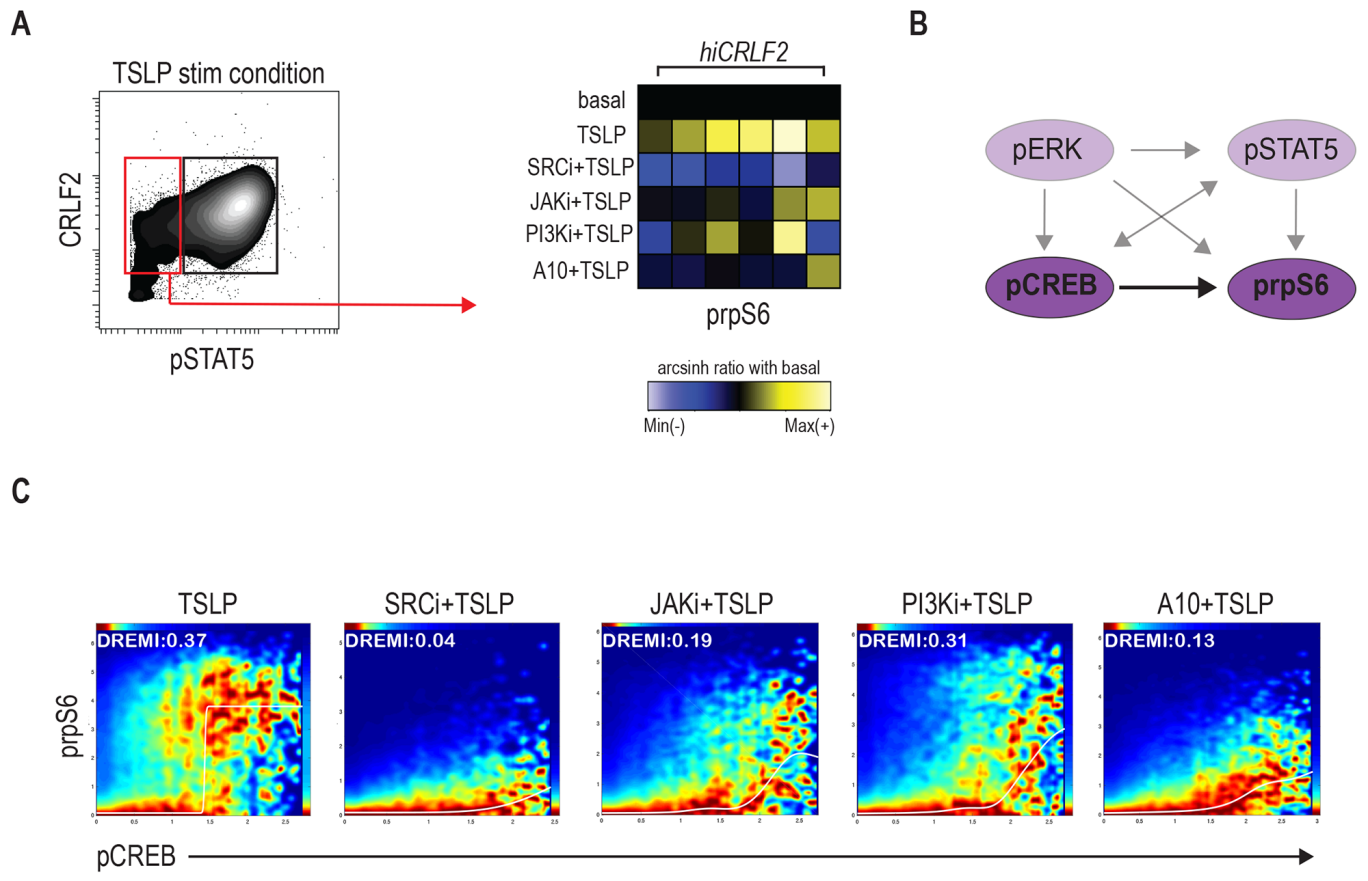
Signaling heterogeneity exists within *hiCRLF2* cells **(A)** Left: contour plot of pSTAT5 activation after TSLP stimulation in a representative *hiCRLF2* case. The red rectangle highlights the pSTAT5 negative cell subset while the black rectangle denotes the pSTAT5 positive cell subset. Right: prpS6 levels in pSTAT5 non-responsive cells. Each column represents a hi*CRLF2* patient and each row the level of prpS6 in the different treatment conditions showed as the arcsinh ratio of the treated condition over baseline levels. **(B)** Graphic representation (dark purple) of the only pairwise connection (pCREB → prpS6) present in the pSTAT5 non-responsive cells assessed by DREMI analysis. The absent connections in this cell subset are shadowed. **(C)** DREVI visualization of conditional density function for pCREB → prpS6 present in the pSTAT5 not responsive cells. Treatment effects on this interaction are showed in the respective condition plots and in each plot are reported the sigmoidal response functions and the DREMI score. SRCi=dasatinib, JAKi=ruxolitinib, PI3Ki=NVP-BEZ235, A10=A10 anti-CRLF2 mAb.

Although aberrant JAK/STAT activation has been observed in *hiCRLF2* BCP-ALL cases, it is clear, as shown in our data and others [15, 26], that the TSLPR network is characterized by complex signaling crosstalk between different pathways. Taking advantage of the high-parameter single-cell resolution of mass cytometry, we identified a subpopulation of CRLF2 positive cells that did not activate pSTAT5 upon TSLP stimulation. This cell subset was found in all our *hiCRLF2* primary samples (Figure 3A, red box) with variable frequency (mean ± SD: 51.36% ± 12.77%). Interestingly, we noticed that the STAT5 non-responsive (STAT5 NR) cells maintained the ability to activate prpS6 to a similar degree as the pSTAT5-responsive cells (mean arcsinh ratio of pSTAT5 responsive 1.31 *vs.* pSTAT5 non-responsive 1.13, p=0.2215). SRC/ABL inhibition was still most effective at inhibiting downstream TSLP signaling in the STAT5 NR cells (p<0.0001). The A10 anti-CRLF2 mAb also showed a strong inhibitory effect (p=0.0081), as compared to JAKi or PI3K/mTORi (p=0.0227 for both agents) (Figure 3A, right heatmap).

Remarkably, by analyzing the conditional dependencies of our network proteins in this cell subset alone, the only connection maintained in these cells was between pCREB and prpS6 (Figure 3B). SRC/ABLi remained the most effective treatment disrupting the pCREB → prpS6 dependency (Figure 3C). Thus, within individual *hiCRLF2* samples, cellular subpopulations are characterized by related but different network structures. This diversity should inform strategies for disruption as demonstrated that SRC/ABL inhibition is most efficient in targeting both cell types. Next, we asked if this network confers survival advantage to *hiCRLF2* expressing cells and if disruption of this network results in apoptosis.

### Optimal disruption of the TSLP-driven signaling network results in decreased cellular survival

Although our previous data demonstrated the effects of single agent kinase inhibition to inhibit activated TSLP signaling, it was not known if this translated into cell death. To assess how the signaling inhibition affects cellular proliferation and to evaluate if combination treatment might be more effective than single agents, we tested different cellular settings *in vitro* (see methods for details).

**Figure 4:**
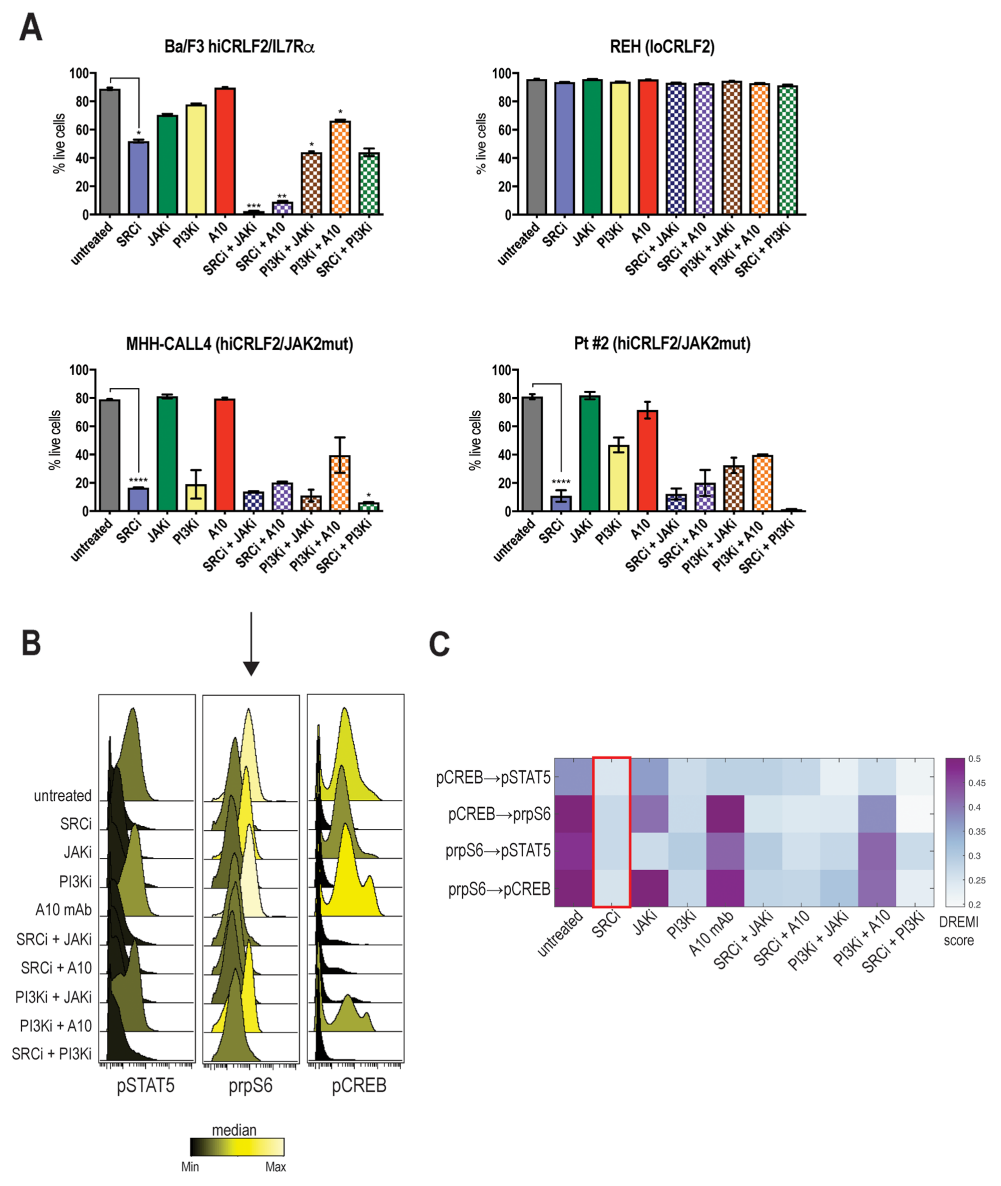
Optimal disruption of the TSLP-driven signaling network results in decreased cellular survival **(A)** Percentage of live cells in single or combined treatments in Ba/F3 *hiCRLF2*-*IL7Rɑ* expressing cells, human REH cells (*loCRLF2*), human MHH-CALL4 cells (*hiCRLF2*/*JAK2* mutated) and in patient-derived primary cells (Pt#2 *hiCRLF2*/*JAK2* mutated). Cells were cultured in presence of TSLP (10 ng/mL) and treated with agents. Combined treatments were compared to the untreated condition and single agent treated cells via one-way ANOVA using Tukey’s post-test for multiple comparisons with ɑ=0.05. The statistics in the figure is reported only for the most effective treatment (SRC/ABLi) compared to either the untreated condition or the combination treatments. ^*^ p<0.5, ^**^ p<0.01, ^***^ p<0.001, ^****^ p<0.0001. **(B)** Histogram overlay of pSTAT5, prpS6 and pCREB levels after treatment of MHH-CALL4 cells *in vitro*. The histograms are colored based on the median levels. **(C)** Heatmap representation of the DREMI score in MHH-CALL4 treated cells showing the pairwise dependencies present in the untreated condition and strongly affected by SRC/ABLi treatment as highlighted by the red box. SRCi=dasatinib, JAKi=ruxolitinib, PI3Ki=NVP-BEZ235, A10=A10 anti-CRLF2 mAb.

In accordance with the signaling data, the SRC/ABLi, dasatinib, was the only agent able to significantly decrease cell viability in all the *hiCRLF2* cells tested (Figure 4A, blue solid bar compared to grey solid bar). By contrast, the JAKi and the anti-CRLF2 mAb did not significantly affect cell viability when used alone (Figure 4A, green and red solid bars); however, when combined with the SRC/ABLi or PI3K/mTORi the effect was significantly higher (Figure 4A, patterned bars). Of note, although the combined JAK/STAT-PI3K/mTOR inhibition resulted in less viable cells than each agent alone, dasatinib alone resulted in superior (see Pt#2) or similar (see MHH-CALL4) levels of apoptosis. Combination treatment with SRC/ABLi plus PI3K/mTORi demonstrated higher levels of apoptosis in MHH-CALL4 and Pt#2. Notably, none of the tested combinations were effective in inducing a pro-apoptotic effect in *loCRLF2* REH cell line (see Figure 4A, upper right), suggesting that the observed effects, were specific to *hiCRLF2* cells.

To examine the effect on the activated TSLP-driven signaling network, we analyzed MHH-CALL4 cells after 96 hours of treatment. Both SRC/ABLi and PI3K/mTORi caused decreased levels of pSTAT5, prpS6 and pCREB (Figure 4B). By contrast, the JAKi was less effective in inhibiting prpS6 and pCREB. DREMI analysis revealed that the strongest dependencies in the TSLP-driven signaling network were between prpS6 and pCREB (pCREB → prpS6 DREMI score 0.50 and prpS6 → pCREB DREMI score 0.56) and prpS6 and pSTAT5 (prpS6 → pSTAT5 DREMI score 0.45) (Figure 4C). The SRC/ABLi could disrupt all of these dependencies (Figure 4C, red box). Thus, inhibiting the TSLP-driven network in *hiCRLF2* cells also decreases cellular survival supporting the importance of this network to enable survival of *hiCRLF2* cells.

### Primary chemotherapy-resistant cells display the CRLF2-driven pro-survival network

Although there is a correlation between *CRLF2* alterations and increased risk of relapse for patients bearing these genetic lesions, the exact role of *CRLF2* overexpression in promoting chemo-resistance is unclear. To determine whether CRLF2 expressing clones persist at early time-points after therapy initiation, we investigated the phenotypic and signaling profiles of cells from MRD samples of 3 additional *hiCRLF2* patients (Pt #13, #14, #15) at Day 8 and Day 15 after initiation of therapy.

**Figure 5:**
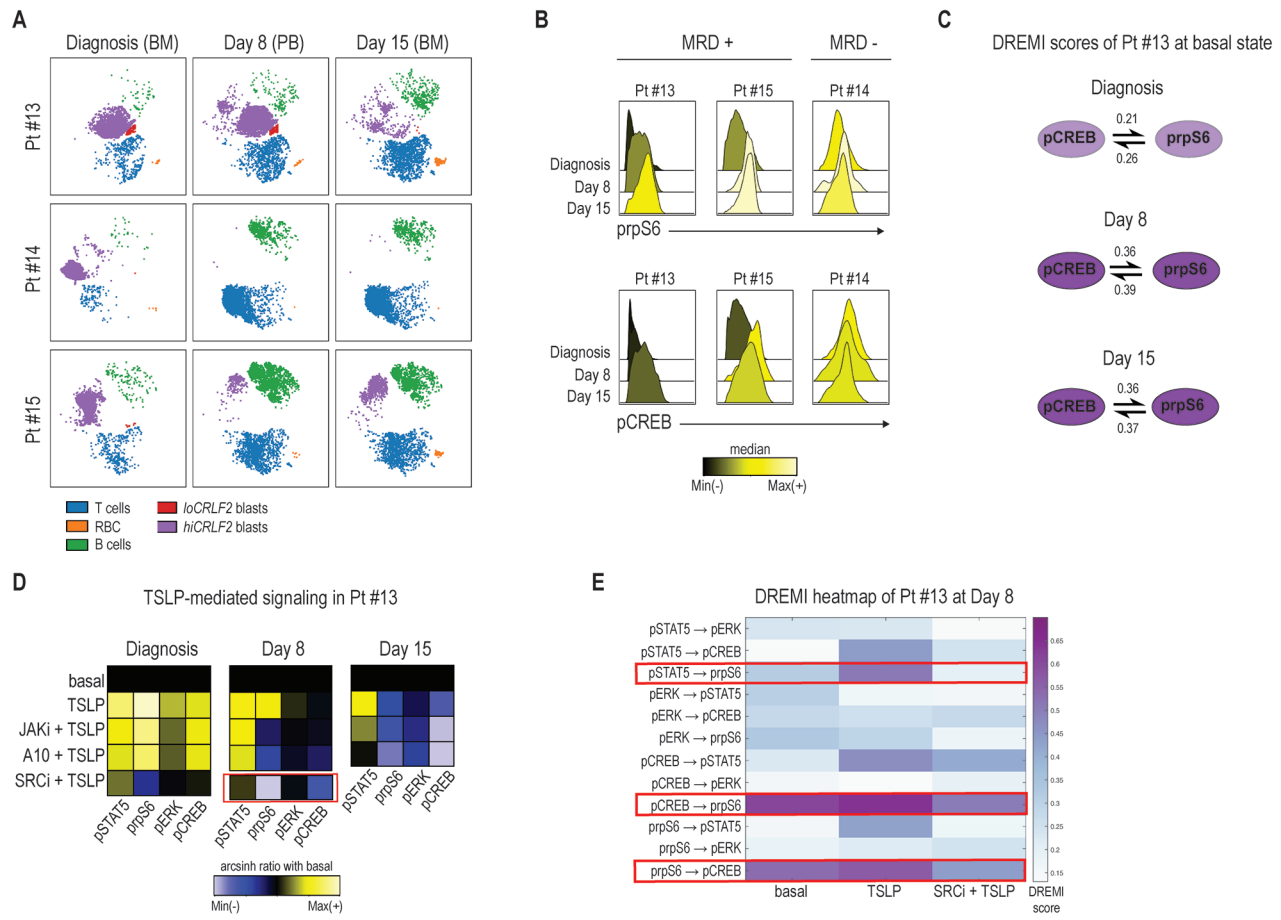
Primary chemotherapy-resistant cells display the CRLF2-driven pro-survival network **(A)** Leukemia response to induction therapy in 3 *hiCRLF2* BCP-ALL primary samples (rows) at 3 different time-points (columns). According to the MRD protocol, at diagnosis and at Day 15, bone marrow samples were analyzed, while, at Day 8, analysis was performed on peripheral blood cells. **(B)** Histogram overlays of prpS6 and pCREB in 3 *hiCRLF2* BCP-ALL primary samples at diagnosis, Day 8 and Day 15 post induction initiation. Histograms are colored based on the raw median of prpS6 and pCREB mean at diagnosis, Day 8 and Day 15. **(C)** Graphic representation of the only pairwise connections (pCREB → prpS6), present at diagnosis (shadowed circles), and enriched at Day 8 and Day 15 (dark purple circles) in Pt#13. The DREMI scores at each time-point and for each connection are reported. **(D)** Heatmaps of pSTAT5, prpS6, pERK and pCREB levels (columns of the heatmaps) in 5 different conditions (rows) at diagnosis, Day 8 and Day 15 in one *hiCRLF2* patient (Pt #13). The heatmap in the SRC/ABLi condition at Day 8 (showed separately) was obtained by a different experiment and the level of the phosphoproteins were normalized based on the basal state of that experiment. Heatmaps are colored with a scale from blue to yellow based on phosphoproteins levels calculated as arcsinh ratio of their levels in the treated conditions (TSLP, JAKi, A10 mAb or SRCi) over baseline levels. **(E)** Heatmap representation of the DREMI score of MRD cells of Pt#13 at Day 8 in the following conditions: basal, TSLP alone, SRCi+TSLP. The red boxes highlight the connections present in the MRD cells and their disruption after treatment with the SRCi. SRCi=dasatinib, JAKi=ruxolitinib, PI3Ki=NVP-BEZ235, A10=A10 anti-CRLF2 mAb.

Mass cytometry detected MRD cells comparably to flow cytometry (FCM) (Supplementary Table 3 and Supplementary Figure 4A-4B). CRLF2 protein expression was maintained at both Day 8 and Day 15 in all *hiCRLF2* samples (Figure 5A, purple population and Supplementary Table 4). Analyzing the activated signaling at Day 8 and Day 15, we showed that patient #14, characterized by a rapid clearance of blast cells by Day 15 (0.08%), demonstrated high basal pCREB and prpS6 at diagnosis and at Day 8 and Day 15, although there were few cells to analyze at these time-points (Figure 5B, MRD- panel). Patients #13 and #15, both clinically classified as high risk with MRD positivity (Table 1), demonstrated increased basal levels of prpS6 and pCREB in MRD cells as compared to the diagnostic sample (Figure 5B, MRD+ panel). For the MRD+ patients (#13 and #15) enough cells were available at MRD time-points to examine the identified CRLF2-driven signaling network by DREMI. In patient (#13), the strongest connections present in the unperturbed MRD cells were between prpS6 and pCREB (Figure 5C and Supplementary Figure 5, left heatmap). The same connection was also present in Pt#15 at diagnosis and persistent in the MRD cells in addition to an association with pERK (prpS6 → pERK and pCREB → pERK) as shown in Supplementary Figure 5 (right heatmap).

To determine if the TSLPR is functional and can be similarly inhibited by the tested TKIs, MRD cells of patient #13 (based on cell availability) were treated with either TSLP alone or in combination with JAKi or A10 or SRC/ABLi (diagnosis, Day 8). Consistent with the diagnostic samples, the SRC/ABLi was the most effective treatment, inhibiting the TSLP-activated signaling, activated prpS6 and pCREB, in the MRD cells at Day 8 (Figure 5D, red box). This was confirmed by DREMI analysis where SRC/ABLi could overcome the TSLP-induced dependencies between pCREB and prpS6 as well as that between pSTAT5 and prpS6 (Figure 5E). Both the A10 mAb and ruxolitinib inhibited TSLP-induced pSTAT5 level at Day 8, and this inhibition increased at Day 15 especially with the A10 mAb (Figure 5D, Day 15 heatmap).

Together, we assessed the feasibility of using CyTOF to detect MRD cells but we were further able to interrogate their signaling network, demonstrating that the network profile present in the *hiCRLF2* cells at diagnosis is enriched in treatment-resistant cells. This network can be disabled by JAK inhibition but a more effective strategy may be inhibition of SRC/ABL in treatment-resistant cells.

## DISCUSSION

*CRLF2* rearrangements and other Ph-like-related kinase alterations are associated with a greater risk of relapse and inferior outcomes in ALL patients [27, 28]. In the last years, several groups have proposed different strategies to target the CRLF2 pathway in pediatric BCP-ALL such as T-cells engineered with a chimeric antigen receptor (CAR) directed against CRLF2 [29], anti-CRLF2 monoclonal antibodies [30, 31] or, more recently, an epigenetic approach using an HDAC inhibitor [32]. However, the role of *CRLF2* alterations and their related signaling in leukemic transformation of B-cells remains only partially understood.

Here, we have further investigated CRLF2 signaling in order to identify signaling nodes downstream of TSLPR that confer a survival advantage to these cells and could be targeted by using kinase inhibitors already used clinically for other subtypes of patients.

To this end, we performed an extensive single-cell profiling of activated phosphoproteins in BCP-ALL leukemic cells in two series of *hiCRLF2* primary samples using mass cytometry, which allows the simultaneous measurement of both surface and signaling molecules in a large number of cells [23, 33]. Furthermore, we also tested the *in vitro* effects on signaling and cell death induction of a selected monoclonal antibody directed against the CRLF2 subunit, and three different KIs (dasatinib, ruxolitinib and NVP-BEZ235) acting at different levels of TSLPR-mediated signaling.

Our findings confirmed and further extended previous data [7, 18, 24] indicating a TSLP-dependent hyper-activation of JAK/STAT, PI3K/mTOR, RAS/MEK pathways, as well as the transcription factor CREB. Interestingly, the phosphorylation of all the analyzed CRLF2 downstream effectors was better exploited by the SRC/ABL inhibitor dasatinib compared to the specific JAK1/2 inhibitor, ruxolitinib, currently tested in phase II clinical study to treat Ph-like ALL patients bearing CRLF2 alterations. Dasatinib effect was strong in all the *hiCRLF2* patients and this could be explained by the activity of dasatinib to act against SRC downstream targets which are also part of the TSLP-driven signaling pathways as demonstrated by Zhong *et al* [7]. Conversely, ruxolitinib activity was mostly restricted to patients bearing also alteration in the JAK2 gene (Pt#1 and Pt2, as described in Table 1). In addition, we observed also a significant *in vitro* activity of the A10 anti-CRLF2 mAb, which could inhibit the TSLP-induced pSTAT5, prpS6 and pERK activation in some *hiCRLF2* patients but was not generally as effective as the SRC/ABLi.

Taking advantage of the single-cell nature of these data and applying bioinformatics tools [25] to investigate signaling dependencies, we demonstrated that the TSLP-induced phosphoproteins act in a coordinated network in which pSTAT5, prpS6, pERK and pCREB are highly connected and the only treatment able to completely disrupt their connections was the SRC/ABL inhibitor, dasatinib. Further, we could also identify a subpopulation of CRLF2 positive cells, present in all the *hiCRLF2* patients, which did not phosphorylate STAT5 upon TSLP stimulation. These cells displayed enhanced TSLP-mediated phosphorylation of rpS6 and a strong signaling connection between prpS6 and pCREB, which was completely inhibited by the SRC/ABLi suggesting that SRC family members, already shown by Zhong *et al.* to be involved in the TSLPR pathway, may be a crucial signaling node that needs to be targeted to affect the coordinated TSLPR signaling.

We next asked how the inhibition of the CRLF2 network affected cell survival. In accordance with the short-term signaling data, we found that the SRC/ABLi dasatinib was most efficient as a single-agent in inducing cell death of *hiCRLF2* cells. Nonetheless, combining dasatinib with a PI3K/mTORi was even more effective in cells bearing not only the *CRLF2* overexpression but also the activating mutation R683G in the *JAK2* gene.

Several studies have questioned the role of CRLF2 aberrations in leukemogenesis due to the presence of CRLF2-positive subclones or loss at the relapse of the disease [34, 35]. When and how this phenomenon occurs is still under debate, however herein we demonstrate that the expression of CRLF2 receptor is maintained in primary MRD cells during early time-points of induction therapy and that, not only are the MRD cells still responsive to TSLP stimulation, but also that the signaling dependencies present at diagnosis, particularly between prpS6 and pCREB, are strengthened in the MRD chemo-resistant cells, proving their importance in the *in vivo* persistence of these cells. The emergence of a stronger dependency between prpS6 and pCREB in the MRD cells compared to that in the total leukemia population at diagnosis is similar to that observed in the STAT5-non-responsive TSLP cells (Figure 3A-3B). Further, this suggests that in the treatment-resistant MRD cells, the strengthening of the relationship between these proteins, as measured by DREMI, may be due to two possibilities: 1) their dependency strengthens with exposure to therapy or 2) the population is becoming more homogeneous such that only the cells with this strong connection survive. This suggests that not all components of the TSLP-driven network confer survival advantages but particular nodes are most critical to target to control drug resistant cells.

Although performed in a very limited series of primary samples, these experiments represent an important proof of principle and feasibility for future applications of mass cytometry to functionally characterize the proteomic profiles of primary MRD cells. In particular, further studies in a larger cohort of MRD samples and also at later time-points of follow-up could help to clarify how CRLF2 expression and its signaling dependencies are affected by the treatment and their role in the relapse of the disease.

In conclusion, our findings demonstrated how applying high-parameter, single-cell analysis to primary *hiCRLF2* BCP-ALL cells enabled a more granular characterization of the TSLP signaling network and its dependencies. We observed heterogeneous cell populations in terms of signaling networks in primary samples yet uncovered the most critical points to target in the network. In doing so, we identified a SRC/ABLi as the most effective to disable the entire signaling network downstream of TSLP which correlated with increased cell death.

## MATERIALS AND METHODS

### Patients and samples

Patient samples were selected based on the overexpression of *CRLF2* gene calculated by RQ-PCR and on the overexpression of CRLF2 protein determined by FCM as previously described [18, 36]. As shown in Table 1, we analyzed a total of 15 diagnostic bone marrow BCP-ALL samples, 9 of which were characterized by an overexpression of the *CRLF2* gene (*hiCRLF2*) and 6 were *CRLF2* low (*loCRLF2*) (assessed by FCM and RQ-PCR). Genetic characterization of patients for JAK2 and IL7R alterations as well as for other BCP-ALL associated aberrations were performed based on samples availability (as further described in Supplementary Materials and Table 1).

Twelve patients (6 *hiCRLF2* and 6 *loCRLF2*) were first analyzed by CyTOF to study the TSLP-related pathways. In addition, 3 further *hiCRLF2* patients were studied during early phases of remission induction therapy (2 samples per patient, collected at Day 8 and at Day 15) and 1 *hiCRLF2* patient out of 9 was used for the *in vitro* agents’ combinations. Further patient’s information is reported as Supplementary Materials.

### Sample preparation, acquisition by CyTOF and data analysis

Samples were processed as previously described [23]. Briefly, viably preserved bone marrow cells were thawed and re-suspended in RPMI 10% FCS, 1% L-glutamine, 1% penicillin/streptomycin, 20 U/mL sodium heparin (Sigma) and 0.025 U/mL benzonase (Sigma). Cells were stained with cisplatin to determine viability [37], rested for 30 minutes at 37°C and then perturbed as described in Supplementary Table 2. Following treatment with TSLP (10 ng/mL for 30 minutes) in the presence or absence of other agents, cells were fixed with formaldehyde (PFA; Electron Microscopy Sciences, Hatfield, PA, USA) to a final concentration of 1.6% for 10 minutes at room temperature. Cells were barcoded using palladium-based labeling reagents as recently described [38], washed using cell staining media (CSM; PBS with 0.5% BSA, 0.02% sodium azide), combined into a single FACS tube and then blocked with purified human Fc receptor binding inhibitor (eBioscience Inc., San Diego, CA) following manufacturer’s instructions. Surface marker antibodies were added yielding 100 μL final reaction volumes and stained at room temperature for 30 minutes. Following staining, cells were permeabilized with 4°C methanol for at 10 min at 4°C, washed twice in CSM and stained with intracellular antibodies cocktail in 50 μL for 30 min at room temperature (Supplementary Table 1). Cells were then stained with 1 mL of 1:5000 191/193Ir DNA intercalator (DVS Sciences, Richmond Hill, Ontario, Canada) diluted in PBS with 1.6% PFA for 20 minutes at room temperature, washed once with CSM and finally with water alone before running on the CyTOF mass cytometer (Fluidigm, Inc., South San Francisco, CA, USA). Normalization of signal intensity loss during the CyTOF run was performed as described before utilizing metal standard beads mixed with the sample during the data acquisition [39]. Mass Cytometry data were analyzed using Cytobank (Cytobank Inc. Mountain View, CA, USA) to generate heatmaps and histogram overlay [40], DREMI and DREVI to reveal pairwise interaction between molecular species [25] and viSNE to visualize high parameters data on two dimensions [41]. Further information are reported in Supplementary material.

### Cell cultures

Human BCP-ALL cell lines, MHH-CALL4 and REH, were purchased from DSMZ (Braunschweig, Germany) and used for *in vitro* drug combination assays. MHH-CALL4 cells overexpress *CRLF2* via IGH@-CRLF2 translocation and bear a JAK2 I682F mutation. REH cells are *CRLF2* wild type and they were used as negative control. Cells were grown in RPMI medium supplemented with 10-20% fetal bovine serum (FBS), 1% L-glutamine and 1% penicillin/streptomycin at 37°C in humidified air with 5% CO_2_. The murine pro-B cell line Ba/F3-hTSLPR, provided by Prof. Shai Izraeli, is derived from Ba/F3 cells transduced with a retrovirus containing human CRLF2 and IL-7Rɑ genes to stably express on their surface a functional human TSLP complex, as previously described [16]. Ba/F3-hTSLPR cells were maintained in RPMI medium supplemented with 10% FBS, 1% penicillin-streptomycin, 1% L-glutamine and 2% interleukin-3 at 37°C in a 5% CO_2_ incubator. Assessment of cell death assays was carried out by FACS analysis (see Supplementary Materials).

### Drugs and antibodies

SRC/ABLi (dasatinib), JAKi (ruxolitinib) and PI3K/mTORi (NVP-BEZ235) were purchased from LC Laboratories^®^ (www.LCLabs.com). The three anti-CRLF2 monoclonal antibodies (clones A10, A11 and G7) were provided by MRCT Centre of Therapeutic Discovery of Leicester under MTA (see Supplementary Materials for further details).

### Statistical analysis

Unpaired two-sided Student’s *t*-test was used to analyze the statistically significant differences between the *hiCRLF2* and *loCRLF2* phosphoprotein groups. Statistical analyses of phosphoprotein expression levels after treatment, calculated as arcsinh ratio in treated (treatment + TSLP) versus untreated condition (TSLP alone), were performed with one-way ANOVA test followed by Dunn’s test for multiple comparisons with α=0.05.

One-way ANOVA test, followed by Tukey’s test for multiple comparisons, was also applied to the *in vitro* cell death assay to evaluate the cell viability in treated *vs* non-treated condition, or in the combined *vs* single treatment. A p value ≤ 0.05 was considered statistically significant. All statistical analyses were performed with GraphPad Prism v 6.0 software (GraphPad, La Jolla, CA, USA). ^*^ p<0.5, ^**^ p<0.01, ^***^ p<0.001, ^****^ p<0.0001.

## SUPPLEMENTARY MATERIALS FIGURES AND TABLES


