# Supplementary material for: SRC/ABL inhibition disrupts CRLF2-driven signaling to induce cell death in B-cell acute lymphoblastic leukemia
Source: Oncotarget. 2018 May 1;9(33):22872–85. doi: 10.18632/oncotarget.25089 (PMC5955419; doi:10.18632/oncotarget.25089)
Supplement: Supplementary file 1 [file oncotarget-09-22872-s001.pdf]

SRC/ABL inhibition disrupts CRLF2-driven signaling to induce cell death in B-cell acute lymphoblastic leukemia

SUPPLEMENTARY MATERIALS

Patients

Diagnostic leukemia samples were obtained, under informed consent, from I-BFM trials (AIEOP-ALL 2000 and AIEOP-ALL 2009 protocols) at the Pediatric Clinic of University Milano-Bicocca (Monza, Italy) and cryopreserved at the M. Tettamanti Research Center (Monza, Italy). Investigation was conducted in accordance with the Declaration of Helsinki and approved by the authors' institutional review boards. Patients were classified as standard risk (SR), intermediate risk (IR), high risk (HR) for MRD and final risk based on diagnostic clinical data [1]. In particular the final risk is assigned based on prednisone response at Day 8, white blood cell counts and MRD risk as previously described [1].

Screening of CRLF2 expression, IL-7, JAK2 alterations and Ph-like status

CRLF2 overexpression, *P2RY8-CRLF2* fusion and *IL-7* gene alterations were analyzed as previously described [2]. Briefly, relative gene expression (indicated as fold change) was quantified by the 2-DDCt method. For *CRLF2* expression, the DDCts were calculated by subtracting to the DCt of each sample the median of the DCt of a published cohort of 464 BCP-ALL patients enrolled in Italy in the AIEOP-BFM ALL 2000 study from February 2003 to July 2005 [2]. Patients were considered *CRLF2* overexpressed when the relative gene expression was 20-fold above the median. Patients were further characterized for *JAK2* alterations by High Resolution Melting (HRM) and for other BCP-ALL associated aberrations by Multiplex Ligation-dependent Probe Amplification (MLPA; SALSA MLPA P335-A3 ALL-IKZF1 probemix, MRC-Holland, Amsterdam, The Netherlands) according to the manufacturer's instruction [3, 4].

Gene expression profile (GEP) analysis was performed for *Ph-like* candidate patients (Pt #4, 5, 6 and 15) using the HG U133 Plus 2.0 platform (Affymetrix, Santa Clara, USA). The gene expression profiles of the samples were classified using the Diagnostic Classifier (DC) model [5, 6] that identifies 17 classes of acute and chronic leukemia (both acute myeloid leukemia and acute lymphoblastic leukemia). According to the DC model the analyzed samples resulted as ALL samples without know aberrations (DC class 8) but with a tie for ALL samples with t(9;22) (DC class 3), indicating a classification as *Ph-like* for all the analyzed patients. Patients #2 and 13 had IGH@-CRLF2 translocation and they can be considered as *Ph-like* according to the literature [7, 8].

Detection of IGH@-CRLF2 translocation

IGH@-CRLF2 translocation was determined in *CRLF2* over-expressed patients, of whom fixed cells from bone marrow at diagnosis were available, by fluorescence *in situ* hybridization (FISH) in interphase nuclei using CRLF2 Breakapart Probe (Cytocell Ltd, Cambridge, UK). Analyses were carried out using a Zeiss Axio Imager Z2 fluorescent microscope (Carl Zeiss AG Corporate, Oberkochen, Germany) and ISIS software (MetaSystems GmbH, Altlussheim, Germany). For each case 150/200 interphase nuclei were scored.

Antibody production

Five SJL/J mice were inoculated of recombinant human CRLF2 Fc chimeric protein (R&D Systems - 981-TR-050) using 100 µg via intraperitoneal injection of CRLF2 on days 1 and 15 with a final intravenous boost injection of 50 µg on day 29. Mice were culled using a humane method (on day 43) and the spleens surgically removed and maintained in sterile RPMI media (GIBCO 31870-025). The animal work was contracted to ENVIGO and performed in line with the EU directive on animals used for scientific purposes (Directive 2010/63/EU). Splenocytes were prepared using gentle MACS dissociator (Miltenyi Biotech 130-093-235) followed by passage through a 40 µm mesh cell strainer (Corning 352340) into sterile media, cells were pelleted by centrifugation at 400 G for 8 minutes, red blood cell lysis was performed by incubating with 20 volumes of 1 x ACK buffer (155mM NH_4Cl, 1mM $KHCO_3$ and 0.1mM EDTA) at room temperature for 5 mins, repeated twice. The resultant cells were then resupended in the cell fusion kit buffer (Genomeone-CF) and fused with NS0 myeloma cells using the HJV-envelope cell fusion kit to produce CLRF2 positive hybridomas (as per manufacturer's instructions). Clones were selected and picked in semi solid media using the clonepix FL (Molecular Devices) following manufacturer's instructions. CRLF2 positive clones were expanded into serum free cultures using hybridoma SFM media (Invitrogen, cat 12045076). Antibodies were affinity purified using Protein G HP (GE, cat 17-0405-03) followed by a size exclusion on a HiLoad 16/600 superdex 200pg column (GE, cat: 28-9893-35) using an AKTA express system (GE, cat: 18664501).

Hybridoma cell lines growth and antibody purification

The hybridoma cell lines were first grown in RPMI 'recovery medium' (GIBCO 31870-025) supplemented with 1x GlutaMAX (GIBCO 35050-038), 100 Units/mL penicillin and 100 μg/mL streptomycin (1:100 of PenStrep solution from GIBCO 15140-122), 1M HEPES (GIBCO 15630-056), 3.8 mM oxaloacetic acid **(**Sigma O7753**),** 0.45 mM sodium pyruvate (Sigma 58636) and 0.2 Units/mL of insulin (Sigma 19278). Cultures were established from cryopreserved hybridomas (A10 and H3) using 1×10^7 cells and maintained, as standard for suspension cell lines, until a doubling time of 2-4 days was reached. The medium was then gradually replaced at a 50:50 ratio for at least 4 weeks by CD Hybridoma Medium (GIBCO 11279-023) supplemented with 3.8 mM oxaloacetic acid (Sigma O7753), 0.45 mM sodium pyruvate (Sigma 58636) and 0.2 Units/mL Insulin (Sigma 19278), as before, and 1 × Cholesterol (GIBCO 12531018). The cultures were expanded until at least 1L of cells was obtained. Cells were then left for 4-6 weeks to 'over-grow' and to accumulate secreted monoclonal antibody. Upon cells removal by centrifugation at 230 G for 5 minutes at 4°C, the medium was harvested and filtered using with a 0.22 μm polyethersulfone stericup-GP vacuum filter system (Millipore SCGPU11RE). The resulting supernatant was then stored at 4°C until purification was performed. Monoclonal antibodies were purified at the Immunopharmacology Lab at Humanitas Research Center (Milan, Italy) by using fast protein liquid chromatography (FPLC).

Mass cytometry data analysis

Single-cell de-convolution algorithm was used as previously described [9, 10], giving a FCS file for each barcode population. All FCS files were analyzed and graphs generated using Cytobank (Cytobank.org; Cytobank Inc. Mountain View, CA). Data were transformed using the inverse hyperbolic sine (arcsinh) equation with a cofactor of 5, and the ratio of phosphoprotein levels in treated conditions (TSLP and/or drugs) over baseline levels was calculated. The heatmaps were colored using a colorimetric scale to depict relatively increased (yellow) or decreased (blue) phosphoproteins, in the treated conditions compared to the levels of the proteins at the basal state (indicated in black). In the MRD analysis, live cells (DNA+, cPARP-) were clustered based on the expression of 11 antigens typical of T, B and red blood cell (RBC) lineages: CD45, CD19, CD10, CD20, CD3, CD235-CD61, intracellular immunoglobulin heavy chain (IgHi), surface IgH (IgHs), CD34, and CD38 using viSNE, powered by Cytobank, to visualize them as previously described [11].

To perform conditional-Density Resampled Estimate of Mutual Information (DREMI) analysis and conditional-Density Rescaled Visualization (DREVI), we sampled up to 15,000 blast cells from each patients at diagnosis, up to 5,000 blast cells from MRD time-points and up to 40,000 live cells from cell line experiment. The strengths of pairwise signaling interactions were calculated in each patient/condition separately or in pooled samples (as shown in Figure 2C) using a MATLAB-based software *simpledremi* (www.c2b2.columbia.edu/danapeerlab/html/dremi/html) as previously described [12].

Drug combinations assay in human BCP-ALL cell lines and one patient-derived primary cells co-coltured with human bone marrow stromal cells

Ba/F3-hTSLPR, MHH-CALL4 and REH cells were seed at concentration of 4^*10^5 cell/mL in 48-well plates (Ba/F3-hTSLPR) and 5^*10^5 cell/mL in 24-well plates (MHH-CALL4 and REH). Cytotoxicity assay were performed after 96 hours of treatment with A10 anti-CRLF2 mAb and/or kinase inhibitors alone and in combinations, by using GFP CERTIFIED® Apoptosis/Necrosis detection kit (Enzo Life Science, Inc., Lausen, Switzerland) following the manufacturer instructions.

Primay cells from one *hiCRLF2* BCP-ALL patient (Pt #2) were co-cultured *in vitro* with a layer of human bone marrow stromal (HBMS) cells [13], kindly provided by Dr. Dario Campana (Department of Paediatrics, National University of Singapore). Co-cultures of primary blast cells and HBMS cells were performed as previously described [14, 15]. Briefly, HBMS cells were seed at concentration of 2^*10^4 cells in 96 wells plate in RPMI-1640 medium with 10% FBS, 10^{-6} mol/l hydrocortisone (Sigma Chemicals, St. Louis, MO, USA), 1% L-glutamine, 1% penicillin-streptomycin. After 3/4 days of culture, when a confluent stromal layer was seen, 2^*10^5 primary cells were resuspended in 200 μL of AIM-V medium with TSLP 10 ng/mL (Life Technologies, Carlsbad, CA, USA) and seeded in 96 wells and incubated at 37°C in 5% of CO_2.

After 30 minutes of culture, cells (cell lines or primary cells) were treated with the A10 anti-CRLF2 mAb and/or kinase inhibitors alone and in combinations at the following concentrations: dasatinib 0.5 μM; ruxolitinib 0.5 μM; NVP-BEZ235 1μM; A10 anti-CRLF2 mAb 20 μg/mL for 72 hours (primary cells) or 96 hours (cell lines) and apoptosis was assessed at FACS by using GFP CERTIFIED® Apoptosis/Necrosis detection kit (Enzo Life Science, Inc., Lausen, Switzerland) following manufacturer instructions. The experiments were performed in triplicate.

Flow cytometry screening of CRLF2 over-expressed patients and MRD detection

The overexpression of the CRLF2 protein was investigated at diagnosis by standard multiparametric flow cytometry (FCM) on fresh samples processed within 24 hours from collection as previously described [16]. Patient's cells were stained with the following combination of mAbs: CRLF2PE/CD45PerCP/CD19APC/CD10PE-cy7/CD7ECD and 30,000 total events were acquired on a FACScanto™ flow cytometer and analyzed with Diva™ software (Becton Dickinson). Measurement of CRLF2 expression was calculated as % of positive cells in the blast population by setting the histograms marker exactly at the right end of normal residual lymphocytes peak. The threshold for antigen positivity was set at ≥10% [16]. The MRD detection by FCM was performed based on a standardized protocol evolved from a previously described method [17].

REFERENCES

1. Conter V, Bartram CR, Valsecchi MG, Schrauder A, Panzer-Grümayer R, Möricke A, Aricò M, Zimmermann M, Mann G, De Rossi G, Stanulla M, Locatelli F, Basso G, et al. Molecular response to treatment redefines all prognostic factors in children and adolescents with B-cell precursor acute lymphoblastic leukemia: results in 3184 patients of the AIEOP-BFM ALL 2000 study. Blood. 2010; 115:3206–14. https://doi.org/10.1182/blood-2009-10-248146.
2. Palmi C, Vendramini E, Silvestri D, Longinotti G, Frison D, Cario G, Shochat C, Stanulla M, Rossi V, Di Meglio a M, Villa T, Giarin E, Fazio G, et al. Poor prognosis for P2RY8-CRLF2 fusion but not for CRLF2 over-expression in children with intermediate risk B-cell precursor acute lymphoblastic leukemia. Leukemia. 2012; 26:2245–53. https://doi.org/10.1038/leu.2012.101.
3. Palmi C, Lana T, Silvestri D, Savino A, Kronnie G Te, Conter V, Basso G, Biondi A, Valsecchi MG, Cazzaniga G. Impact of IKZF1 deletions on IKZF1 expression and outcome in Philadelphia chromosome negative childhood BCP-ALL. Reply to "Incidence and biological significance of IKZF1/Ikaros gene deletions in pediatric philadelphia chromosome negative and philadelphia. Haematologica. 2013; 98. https://doi.org/10.3324/haematol.2013.099077.
4. Palmi C, Valsecchi MG, Longinotti G, Silvestri D, Carrino V, Conter V, Basso G, Biondi A, Te Kronnie G, Cazzaniga G. What is the relevance of Ikaros gene deletions as a prognostic marker in pediatric philadelphia-negative B-cell precursor acute lymphoblastic leukemia? Haematologica. 2013; 98:1226–31. https://doi.org/10.3324/haematol.2012.075432.
5. Haferlach T, Kohlmann A, Wieczorek L, Basso G, Te Kronnie G, Béné MC, De Vos J, Hernández JM, Hofmann WK, Mills KI, Gilkes A, Chiaretti S, Shurtleff SA, et al. Clinical utility of microarray-based gene expression profiling in the diagnosis and subclassification of leukemia: report from the international microarray innovations in leukemia study group. J Clin Oncol. 2010; 28:2529–37. https://doi.org/10.1200/JCO.2009.23.4732.
6. Bresolin S, Zecca M, Flotho C, Trentin L, Zangrando A, Sainati L, Stary J, De Moerloose B, Hasle H, Niemeyer CM, Te Kronnie G, Locatelli F, Basso G. Gene expression-based classification as an independent predictor of clinical outcome in juvenile myelomonocytic leukemia. J Clin Oncol. 2010; 28:1919–27. https://doi.org/10.1200/JCO.2009.24.4426.
7. Panzer-Grümayer R, Köhrer S, Haas OA. The enigmatic role (s) of P2RY8-CRLF2. Oncotarget. 2017; 8:96466–96467. https://doi.org/10.18632/oncotarget.22098.
8. Roberts KG, Li Y, Payne-Turner D, Harvey RC, Yang Y-L, Pei D, McCastlain K, Ding L, Lu C, Song G, Ma J, Becksfort J, Rusch M, et al. Targetable kinase-activating lesions in Ph-like acute lymphoblastic leukemia. N Engl J Med. 2014; 371:1005–15. https://doi.org/10.1056/NEJMoa1403088.
9. Zunder ER, Finck R, Behbehani GK, Amir E-AD, Krishnaswamy S, Gonzalez VD, Lorang CG, Bjornson Z, Spitzer MH, Bodenmiller B, Fantl WJ, Pe'er D, Nolan GP. Palladium-based mass tag cell barcoding with a doublet-filtering scheme and single-cell deconvolution algorithm. Nat Protoc. Nature Publishing Group; 2015; 10:316–33. https://doi.org/10.1038/nprot.2015.020.
10. Bodenmiller B, Zunder ER, Finck R, Chen TJ, Savig ES, Bruggner R V, Simonds EF, Bendall SC, Sachs K, Krutzik PO, Nolan GP. Multiplexed mass cytometry profiling of cellular states perturbed by small-molecule regulators. Nat Biotechnol. 2012; 30:858–67. https://doi.org/10.1038/nbt.2317.
11. Amir ED, Davis KL, Tadmor MD, Simonds EF, Levine JH, Bendall SC, Shenfeld DK, Krishnaswamy S, Nolan GP, Pe'er D. viSNE enables visualization of high dimensional single-cell data and reveals phenotypic heterogeneity of leukemia. Nat Biotechnol. Nature Publishing Group; 2013; 31:545–52. https://doi.org/10.1038/nbt.2594.
12. Krishnaswamy S, Spitzer MH, Mingueneau M, Bendall SC, Stone E, Pe D, Nolan GP. Conditional Density-based Analysis of T cell signaling in Single Cell Data. Science (80-). 2014; 346. https://doi.org/10.1126/science.1250689. Conditional.
13. Mihara K, Imai C, Coustan-Smith E, Dome JS, Dominici M. Development and functional characterization of human bone marrow mesenchymal cells immortalized by enforced expression of telomerase. Br J Haematol. 2003; 120:846–9.

14. Manabe BA, Coustan-smith E, Behm FG, Raimondi SC, Campana D. Bone marrow-derived stromal cells prevent apoptotic cell death in B-lineage acute lymphoblastic leukemia. 1992; 79:2370–7.
15. Todisco E, Gaipa G, Biagi E, Bonamino M, Gramigna R, Introna M, Biondi A. CD40 ligand-stimulated B cell precursor leukemic cells elicit interferon-γ production by autologous bone marrow T cells in childhood acute lymphoblastic leukemia. Leukemia. 2002; 16:2046–54. https://doi.org/10.1038/sj.leu.2402672.
16. Bugarin C, Sarno J, Palmi C, Savino AM, Te Kronnie G, Dworzak M, Schumich A, Buldini B, Maglia O, Sala S, Bronzini I, Bourquin J-P, Mejstrikova E, et al. Fine tuning of surface CRLF2 expression and its associated signaling profile in childhood B cell precursor acute lymphoblastic leukemia. Haematologica. 2015; 1003324:229–32. https://doi.org/10.3324/haematol.2014.114447.
17. Gaipa G, Cazzaniga G, Valsecchi MG, Panzer-Gr??mayer R, Buldini B, Silvestri D, Karawajew L, Maglia O, Ratei R, Benetello A, Sala S, Schumich A, Schrauder A, et al. Time point-dependent concordance of flow cytometry and real-time quantitative polymerase chain reaction for minimal residual disease detection in childhood acute lymphoblastic leukemia. Haematologica. 2012; 97:1586–93. https://doi.org/10.3324/haematol.2011.060426.
18. Basso G, Veltroni M, Valsecchi MG, Dworzak MN, Ratei R, Silvestri D, Benetello A, Buldini B, Maglia O, Masera G, Conter V, Arico M, Biondi A, et al. Risk of relapse of childhood acute lymphoblastic leukemia is predicted by flow cytometric measurement of residual disease on day 15 bone marrow. J Clin Oncol. 2009; 27:5168–74. https://doi.org/10.1200/JCO.2008.20.8934.

Supplementary Table 1: Mass cytometry antibody reagents

Protein	Clone	Manufacturer	Metal Isotope	Surface or Intracellular staining
4EBP1(pT36/T45)	M31-16	Cell Signaling Technology	Nd144	I
Akt (pS473)	193H12	Cell Signaling Technology	Tb159	I
CD10	HI10a	Biolegend	Gd156	S
CD123	6H6	Biolegend	Eu151	S
CD127	HCD127	Biolegend	Dy162	S
CD16	3G8	BD Biosciences	Yb171	S
CD179a	HSL96	Biolegend	Sm149	I
CD179b	HSL11	Biolegend	Gd158	I
CD19	H1B19	Biolegend	Nd142	S
CD20	2H7	Biolegend	Sm147	S
CD22	HIB22	Biolegend	Nd143	S
CD235	HIR2	Biolegend	In113	S
CD24	ML5	Biolegend	Gd160	S
CD33	HIM3-4	BD Biosciences	Yb171	S
CD34	8G12	BD Biosciences	Nd148	S
CD38	HIT2	Biolegend	Er168	S
CD43	CD43-10G7	Biolegend	Er167	S
CD45	HI30	Biolegend	In115	S
CD58	TS2-9	Biolegend	Tm169	S
CD61	VI-PL2	BD Biosciences	In113	S
CD79b	CB3-1	Biolegend	Nd146	S
cPARP	F21-852	BD Biosciences	La139	I
CREB (pS133)	87G3	Cell Signaling Technology	Yb176	I
CRLF2	1B4	Biolegend	Dy161	S
Erk1/2 (pT202/pY204)	D13	Cell Signaling Technology	Yb173	I
HLA-DR	L243	Biolegend	Yb174	S
IgHi	polyclonal	Invitrogen	Eu153	I
IgHs	polyclonal	Invitrogen	Lu175	S
Ikaros (pS63)	STA9	Biolegend	Gd155	I
IgL kappa	MHK-49	Biolegend	Sm154	I
IgL lambda	MHL-38	Biolegend	Sm154	I
Ki67	B56	BD Biosciences	Sm152	I
Pax5	1H9	eBioscience	Ho165	I
PLCg2 (pY759)	K86-689.37	BD Biosciences	Pr141	I
RAG1	D36B3	Cell Signaling Technology	Dy163	I
rpS6 (pS235/pS236)	N7-548	BD Biosciences	Yb172	I
STAT5 (pY694)	47	BD Biosciences	Nd150	I
TdT	E17-1519	BD Biosciences	Dy164	I
ZAP70/SYK (pY319/ pY352)	17a	BD Biosciences	Er166	I

Supplementary Table 2: Treatment conditions

Conditions	Concentration	Time of treatment (minutes)
basal		
PVO_4	125 µM	15’
TSLP	10 ng/mL	30’
dasatinib + TSLP	500 nM + 10 ng/mL	30’ + 30’
ruxolitinib + TSLP	250 nM + 10 ng/mL	30’ + 30’
NVP-BEZ235 + TSLP	1 µM + 10 ng/mL	30’ + 30’
A10 mAb + TSLP	20 ug/mL + 10 ng/mL	30’ + 30’

All treatments were performed after 30 minutes of starvation. The pervanadate (PVO_4) was used as positive control since it can induce maximum phosphorylation of phospho-epitopes.

Supplementary Table 3: Comparison between FCM and CyTOF in the detection of MRD

Patient	Diagnosis		Day 8		Day 15	
	FCM	CyTOF	FCM	CyTOF	FCM	CyTOF
#13	94.0%	88.2%	66.0%	58.5 %	9.1%	10.3 %
#14	85%	95.3	0.03%	0.07%	0.02%	0.08%
#15	86%	85.7%	5.6%	4.4%	12%	13.4%

Supplementary Table 4: Expression of CRLF2 in MRD cells

Patient	% of CRLF2+ blast cells		
	Diagnosis	Day 8	Day 15
#13	75.0%	61.6%	9.6%
#14	90.0%	0.07%	0.06%
#15	73.3%	5.1%	10.7%

Values are percentage of CRLF2 positive cells in MRD gated cells. Proportion of MRD cells in total cells are reported in Supplementary Table 3.

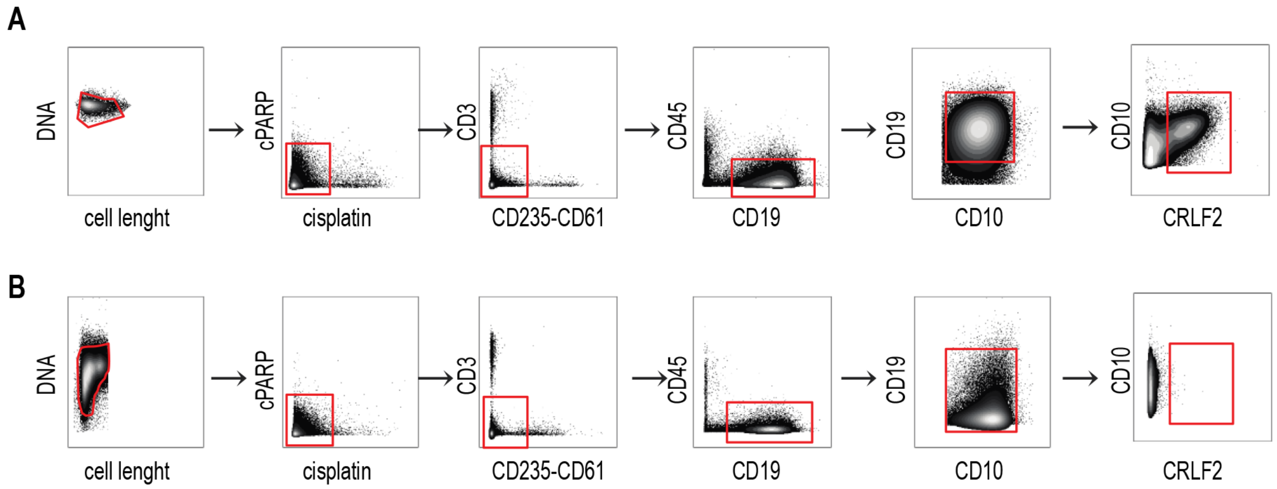

Supplementary Figure 1: Gating strategies applied to detect blast cells in primary samples. (A-B) Hierarchical gating strategy employed to measure CRLF2 surface expression and activated phosphoproteins. Cells, identified on the basis of DNA staining, were distinguished from debris and then gated as viable (cPARP negative/cisplatin negative). T-cells (CD3+), red blood cells (CD235+) and platelets (CD61+) were excluded from the analysis. B-cell blasts were selected as CD45 low-negative/CD10+ and CD19+. Once gated the blast cells, the CRLF2 surface expression was assessed in 15 primary samples. Two representative cases are shown: one *hiCRLF2* (panel A) and one *loCRLF2* (panel B).

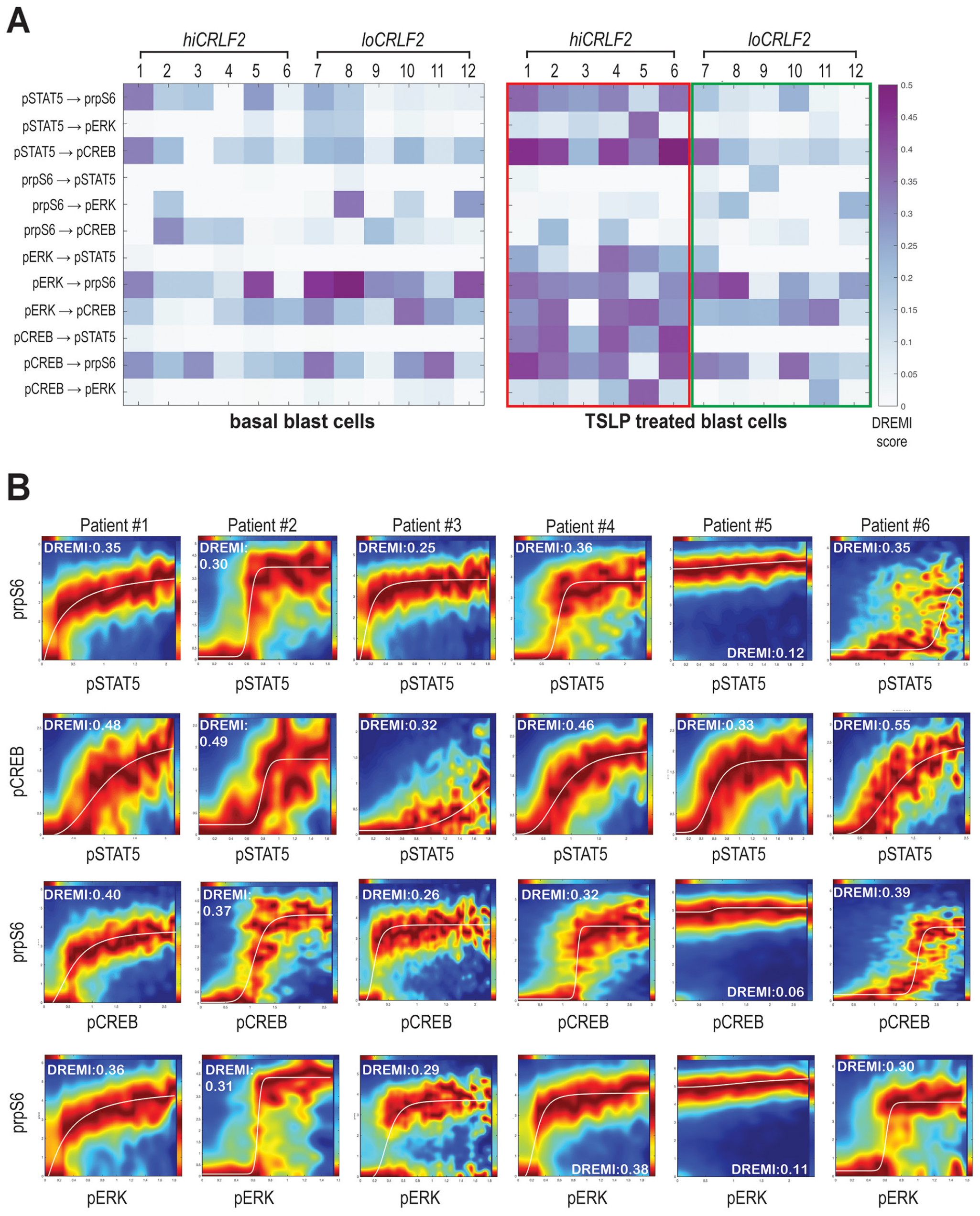

Supplementary Figure 2: DREMI analysis of BCP-ALL primary samples in the basal state and in the TSLP-stimulated state. (A) Heatmaps overview of the DREMI score calculated in the blast cells of 12 BCP-ALL primary samples analyzed (6 *hiCRLF2* and 6 *loCRLF2*) at basal state and after TSLP stimulation (10 ng/mL). The red box highlights the activated connections within prpS6, pSTAT5, pCREB and pERK present after TSLP stimulation in *hiCRLF2* patients compared to the *loCRLF2* patients (green box). On the right is reported the colorimetric scale used to generate the heatmap. **(B)** DREMI analysis and DREVI visualization of the TSLP-induced phosphoptoteins in the six *hiCRLF2* patients. The conditional density functions for pSTAT5 → prpS6 (first row), pSTAT5 → pCREB (second row), pCREB → prpS6 (third row) and pERK → prpS6 (fourth row) are shown. The sigmoidal response functions and the DREMI score are reported in each plot as quantification of the strengths of pairwise signaling relationships within the different networks.

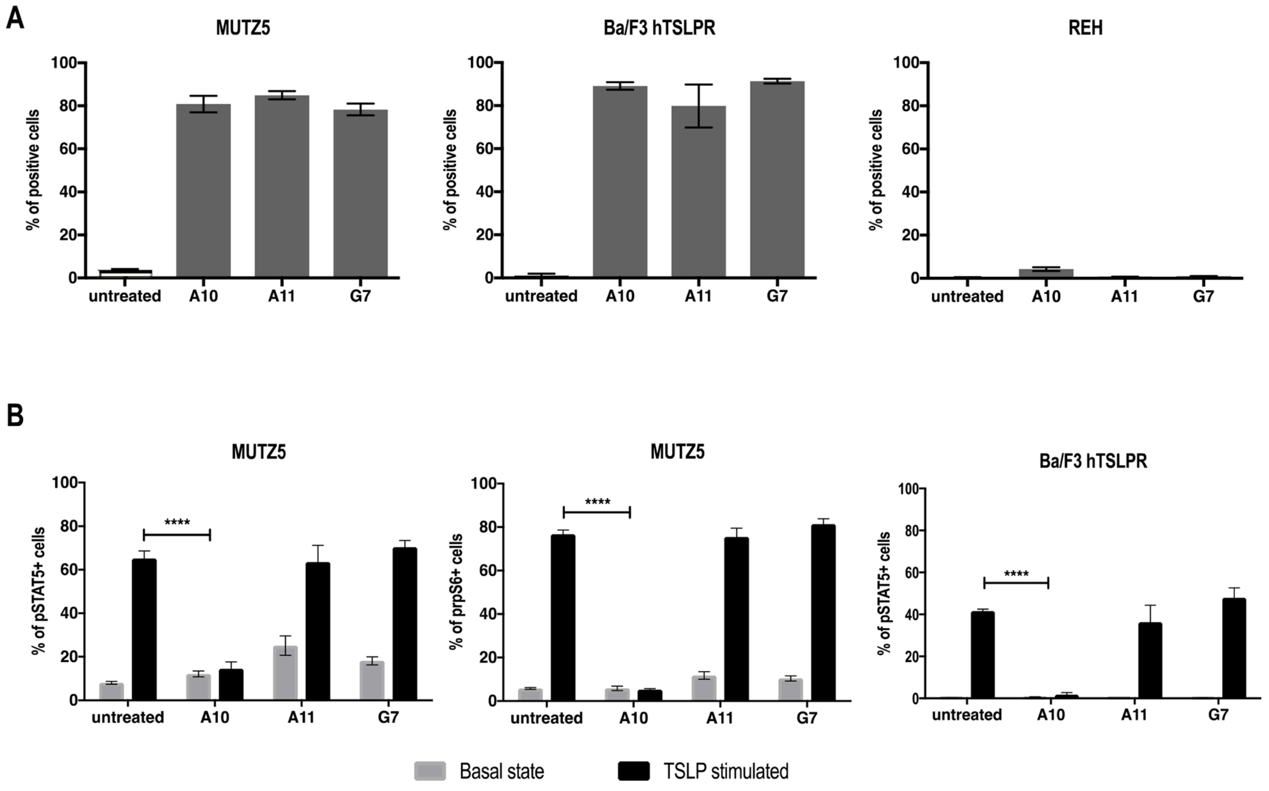

Supplementary Figure 3: Selection of the most promising anti-CRLF2 mAb based on binding and signaling inhibition. **(A)** Column bars showing the percentage of positive cells after 30 minutes treatment with different clones of anti-CRLF2 mAbs (A10, A11, G7) compared to the negative control (untreated cells). The binding is assessed in CRLF2 positive cell line, MUTZ5 and Ba/F3-hTSLPR, and in CRLF2 negative cell line, REH.**(B)** Levels of pSTAT5 (left panel), prpS6 (middle panel) in MUTZ5 cell and pSTAT5 in Ba/F3-hTSLPR (right panel) at basal state (grey columns) and after TSLP stimulation 10 ng/mL (columns bars). Cells were starved for 16 hours in X-vivo medium and then treated with the anti-CRLF2 mAbs for 30 minutes before assessing the levels of the phosphoproteins. Treatment groups were compared to the untreated control via two-way ANOVA with Bonferroni's post-test for multiple comparisons. $^{*}p<0.05$, $^{**}p<0.01$, $^{***}p<0.001$, $^{****}p<0.0001$.

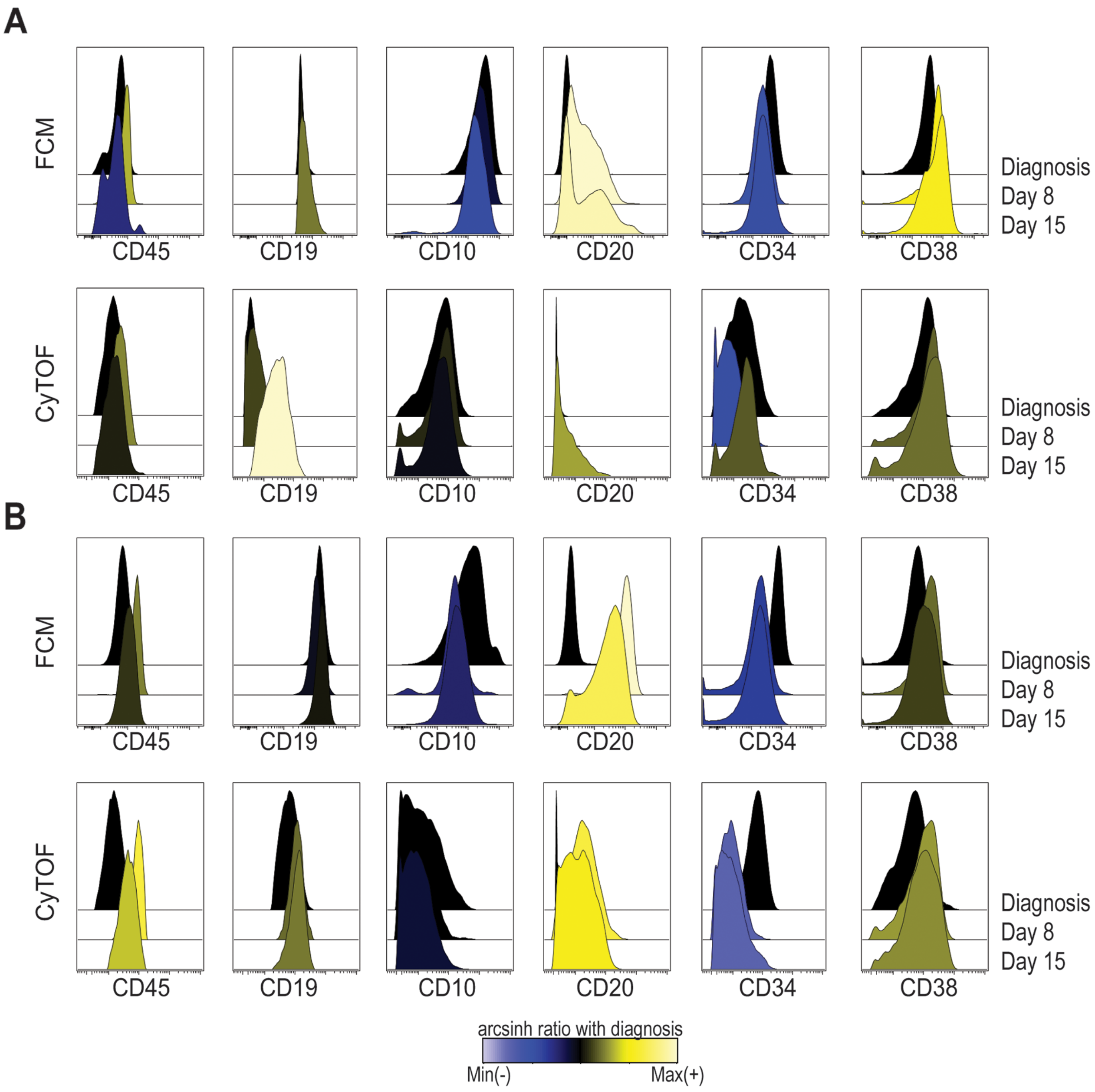

Supplementary Figure 4: MRD immunophenotypic comparison between mass cytometry and flow cytometry. (A-B) Histogram overlay of the expression of the different markers at diagnosis, day 8, and day 15 in 2 (panels A and B) out of 3 *hiCRLF2* analyzed patients. The histograms are colored by statistics (arcsinh ratio of mean at day 8 and day 15 *vs.* diagnosis). Although with different intensities, it is possible to observe a similar trend expression of the immunophenotypic markers widely used for MRD detection [18].

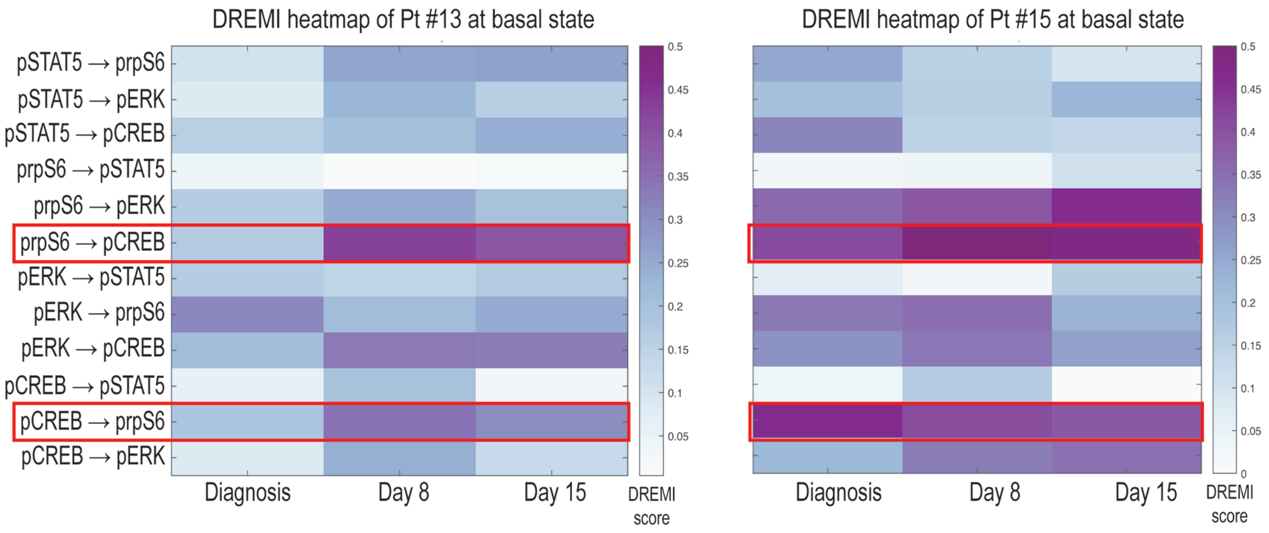

Supplementary Figure 5: DREMI analysis of *hiCRLF2* cells at diagnosis and MRD time-points. Heatmaps overview of the DREMI score calculated in the blasts cells at diagnosis, day 8 and day 15 of Pt#13 (right) and Pt#15 (left). The red boxes highlight the activated connections within prpS6, and pCREB present at diagnosis and enriched in the MRD time points. On the right is reported the colorimetric scale used to generate the heatmap.
